# The Role of Fecal Microbiota Transplantation in Reducing Intestinal Colonization With Antibiotic-Resistant Organisms: The Current Landscape and Future Directions

**DOI:** 10.1093/ofid/ofz288

**Published:** 2019-06-22

**Authors:** Michael H Woodworth, Mary K Hayden, Vincent B Young, Jennie H Kwon

**Affiliations:** 1 Division of Infectious Diseases, Department of Medicine, Emory University School of Medicine, Atlanta, Georgia; 2 Division of Infectious Diseases, Department of Internal Medicine, Rush Medical College, Chicago, Illinois; 3 Division of Infectious Diseases, Department of Internal Medicine, University of Michigan Medical School, Ann Arbor; 4 Division of Infectious Diseases, John T. Milliken Department of Internal Medicine, Washington University School of Medicine, St Louis, Missouri

**Keywords:** antibiotic resistance, antibiotic-resistant organism, fecal microbiota transplantation, hospital epidemiology, microbiome, multidrug-resistant organisms, resistome

## Abstract

The intestinal tract is a recognized reservoir of antibiotic-resistant organisms (ARO), and a potential target for strategies to reduce ARO colonization. Microbiome therapies such as fecal microbiota transplantation (FMT) have been established as an effective treatment for recurrent *Clostridioides difficile* infection and may be an effective approach for reducing intestinal ARO colonization. In this article, we review the current published literature on the role of FMT for eradication of intestinal ARO colonization, review the potential benefit and limitations of the use of FMT in this setting, and outline a research agenda for the future study of FMT for intestinal ARO colonization.

## Teaser

This review summarizes clinical outcomes data for fecal microbiota transplantation (FMT) for antibiotic-resistant organism (ARO) decolonization, addresses limitations in clinical studies of FMT for ARO decolonization, and outlines a clinical research agenda for future study of FMT for this indication.

**Figure 1.**
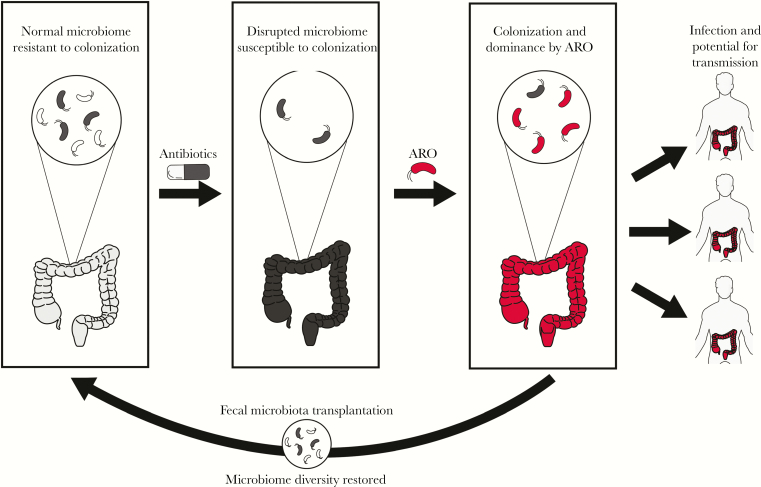
Concept illustration of intestinal microbial diversity as a protective factor against colonization with antibiotic-resistant organisms (AROs), adapted from Halpin et al [25]. Antibiotic exposure can lead to disruption of these community structures and subsequent colonization and dominance by AROs, which may increase risk of infection and transmission to other patients. Fecal microbiota transplantation may reduce risk of ARO colonization and transmission by increasing intestinal microbiome diversity.

The US Centers for Disease Control and Prevention has estimated that each year >2 000 000 patients are infected with antibiotic-resistant organisms (AROs) and 23 000 die of these infections [1]. Infections due to AROs represent an urgent threat to public health and rates of antibiotic resistance are increasing faster than the development of new antimicrobials [2, 3]. The intestinal tract can function as a reservoir for AROs, meaning AROs can be present without causing clinical symptoms [4]. Patients who are colonized with AROs are at risk of ARO infection and ARO transmission to other individuals (Figure 1) [5]. Although aggressive infection prevention interventions can help reduce their spread, these efforts do not control the source of ARO colonization [6, 7].

Multiple studies have used antimicrobials in an attempt to reduce ARO colonization or infection. However, demonstration of improvement in clinical end points has been inconsistent. The use of antimicrobials may also have unintended consequences of selection and expansion of AROs [8–11]. Thus, existing strategies can perpetuate a vicious cycle of increasing antimicrobial use, and pressure for expansion of antimicrobial resistance.

The intestinal microbiome of healthy patients is often characterized as diverse and resistant to ARO colonization. This protection from ARO colonization has bolstered enthusiasm for study of fecal microbiome therapeutics as an antibiotic-sparing approach to address antimicrobial resistance [5]. In the current article, we review published data on the role of fecal microbiota transplantation (FMT) for ARO control, summarize reported clinical outcomes data for the use of FMT to directly reduce ARO colonization, and outline a research agenda for advancing understanding of FMT for this application.

## ARO COLONIZATION AS A COMPLEX PHENOMENON

Studies of ARO colonization have used different definitions of colonization and loss of colonization. This complicates the interpretation of estimates across settings. The detection of even a single isolate with a transmissible resistance mechanism is likely to have important public-health implications. However, the minimum criteria for colonization may lack specificity and in some cases may be overly sensitive. On the other hand, definitions of loss of colonization have also varied and may not be sensitive enough. Documentation with 3 consecutive negative stool or rectal swab cultures is frequently used to define loss of colonization, but some investigators have used a single negative result or ≥2 negative consecutive stool or rectal swab cultures [12, 13]. Further complicating these definitions is the fact that intermittent fecal ARO detection after negative cultures has been described in multiple studies [13–15]. The potential limitations in sensitivity of detection of colonization was further underscored in 1 study with findings suggesting that vancomycin-resistant *Enterococcus* (VRE) colonization was detectable and genotypically similar, as shown by pulse-field gel electrophoresis, even in “cleared” patients 5 years after initial detection [16].

Most published active surveillance studies of intestinal ARO colonization demonstrate themes of prolonged colonization, intermittent periods of shedding, variability in isolate recovery patterns by culture method and by ARO type, and codetection of multiple AROs [14, 17–21]. The duration of colonization also varies by ARO type. The reported median duration of colonization was 306 days (range, 1–1393 days) for VRE in 1 study [14] and 144 days (41–359 days) for multidrug-resistant gram-negative bacteria in another study [17], and the medians for carbapenem-resistant Enterobacteriaceae (CRE) in 2 studies were 165 and 295 days [19, 20]. Compared with patients with a single admission, those readmitted to hospitals or post–acute care facilities have been observed to have variable durations of colonization [20]. The majority of published studies documenting the duration of ARO colonization have investigated outbreak scenarios or patients in acute care or post–acute care facilities, where apparently persistent ARO colonization may be due to ongoing ARO exposures and recolonization [22]. The variability in the natural history of ARO colonization makes decolonization outcomes after FMT challenging to interpret.

Data on the frequency of patient outcomes after ARO colonization are mixed but important to quantify. The development of United States Food and Drug Administration (FDA)–approved treatments for decolonization may rely on improving outcomes such as ARO infection. VRE colonization precedes infection in immunocompromised patients [23]. Isendahl et al [24] reported population-level frequency estimates of bloodstream infection among patients with urine or fecal extended-spectrum β-lactamase (ESBL)–producing Enterobacteriaceae colonization. Of patients with ESBL bloodstream infections, 98.6% had antecedent urine or stool colonization [24]. More work is needed to better determine which patients who are colonized with AROs will become infected and to estimate the number of colonized patients needed to treat to prevent infection, hospitalization, mortality, and other patient-centered outcomes.

## THE HUMAN INTESTINAL MICROBIOME AS A THERAPEUTIC TARGET FOR ARO DECOLONIZATION

Although it is well established that anaerobic bacteria residing in the intestine can limit ARO colonization, the ideal strategy to modify intestinal microbiomes has not been defined. For decades, the association of antibiotic administration and subsequent ARO detection has been understood in part to be an indirect effect mediated by off-target loss of anaerobic taxa as a consequence of antianaerobic antimicrobial activity [17, 18, 24, 26]. This principle was demonstrated by Donskey et al [18] in their prospective surveillance of density of VRE in stool of colonized patients, which showed an expansion of VRE density in stool cultures of patients receiving antianaerobic antibiotic regimens, compared with those not receiving such regimens. Counterintuitively, gram-negative antibiotic treatment has been associated with a doubled risk of bacteremia in ESBL-colonized patients [24]. Similarly, O’Fallon et al [17] noted that two-thirds of patients with persistent multidrug-resistant gram-negative bacterial colonization did not receive antibiotics during their prospective surveillance study, underscoring that factors other than antibiotics also drive colonization.

These observations point to complex interactions between healthy microbiota, AROs, and the host, which have been reviewed elsewhere [27]. Key examples of mechanisms of colonization resistance include resistance to VRE colonization with defined bacterial consortia and with viral and viruslike Toll-like receptor simulation of the antimicrobial peptide Reg3γ [28, 29]. Another established mechanism of colonization resistance is competition between commensals and potential pathogens for dietary and host-derived glycans and metabolites that are nutritional requirements [27]. As mechanisms of colonization resistance continue to be elaborated, FMT is being explored as a method to transfer these identified and unidentified ARO-resistant factors to ARO-colonized patients.

FMT is the process of transplanting stool from a healthy donor to a diseased recipient. Practices similar to FMT have been traced to the Dong-jin dynasty of fourth-century China and reported in contemporary medical literature for treatment of pseudomembranous colitis in 1958 [30, 31]. Since a landmark randomized controlled trial of FMT for treatment of recurrent *Clostridioides difficile* infection (RCDI) was published in 2013, a number of clinical trials have demonstrated cure rates of approximately 90% when repeated FMTs are included [31–35]. FMT has become an important treatment for RCDI and is included in major society guidelines including those produced by the Infectious Disease Society of America and a number of European professional societies [37, 38]. With increasing use of FMT for RCDI, loss of ARO colonization has been increasingly recognized as a collateral benefit in these patients and has been described in increasing numbers of case reports and case series.

## EFFICACY OF INTESTINAL MICROBIOME THERAPIES FOR ARO DECOLONIZATION AMONG PATIENTS WITH RCDI

The use of FMT for RCDI expanded after publication of a Dutch randomized, controlled trial and the decision of the FDA to allow the use of FMT under an enforcement discretion policy in the United States. Some patients treated for RCDI were found to also be colonized with other AROs, and in some these AROs cleared after FMT (Table 1).

**Table 1.** Summary of Published Case Reports and Series Describing Antibiotic-Resistant Organism Decolonization as Secondary Outcome Among Patients Treated With Fecal Microbiota Transplantation for Recurrent *Clostridioides difficile* Infection

Authors	Infection/Colonization Status	Pretreatment	FMT Donor or Product, No. of FMTs, Route	Outcomes	Adverse Events	Duration of Follow-up, mo
Jang et al [39]	RCDI, VRE colonization (n = 1)	Oral vancomycin and intravenous metronidazole	Brother, 2, rectal enema	Clinical RCDI cure; VRE cultures did not clear within time frame of follow-up	None noted	3
Crum-Cianflone et al [40]	RCDI, CR *Pseudomonas*, MDR *Acinetobacter*, CR *Klebsiella,* VRE, MRSA (n = 1)	Oral vancomycin	Sister, 1, colonoscopy	Clinical RCDI cure; reduced clinically indicated cultures obtained, 4/11 cultures with AROs vs with 12/24 before FMT No episodes of sepsis in post-FMT period	None noted	24
Stripling et al [41]	RCDI, recurrent VRE bacteremia, UTIs (n = 1)	Oral vancomycin	Spouse, 1, nasogastric tube	No further VRE infections or RCDI in year after FMT	None noted	12
García-Fernández et al [42]	RCDI, VIM-1–producing *K. oxytoca* colonization (n = 1)	Oral vancomycin	Son, 1, colonoscopy	Clinical RCDI cure; VIM-1 *Klebsiella oxytoca* culture and PCR negative at 6 wk and 6 mo	Constipation at 6 wk	6
Dubberke et al [12]	RCDI, VRE colonization (n = 11)	Variable treatment for RCDI	RBX2660, 1–2, rectal enema	8/11 VRE culture negative at 1–6 mo	Diarrhea, flatulence, abdominal pain and cramping, constipation	6
Tariq et al [43]	RCDI, RUTI (n = 8)	Variable treatment for CDI	Healthy donor, doses not reported, colonoscopy	Reduction in UTI frequency; overall improved antibiotic susceptibility of uropathogens	None noted	12
Wang et al [44]	RCDI, RUTI (n = 1)	Oral vancomycin	Healthy donor, 1, colonoscopy	Clinical CDI cure; no UTI recurrence at 25 mo	None noted	25

Abbreviations: ARO, antibiotic-resistant organism; CDI, *Clostridioides difficile* infection; CR, carbapenem-resistant; FMT, fecal microbiota transplantation; MDR, multidrug-resistant; MRSA, methicillin-resistant *Staphylococcus aureus*; PCR, polymerase chain reaction; RCDI, recurrent CDI; RUTI, recurrent urinary tract infection; UTI, urinary tract infection; VIM-1, Verona integron–encoded metallo-β-lactamase 1; VRE, vancomycin-resistant *Enterococcus* spp.

Stripling et al [41] described the decreased intestinal relative abundance of VRE in a heart-kidney transplant recipient with RCDI and recurrent VRE infections treated with FMT. The potential confounding of stopping vancomycin used for RCDI treatment before FMT and decreased VRE relative abundance in stool was acknowledged as a limitation [41]. However, an increase in the relative abundance of genera that were differentially abundant in donor stool, such as *Blautia, Akkermansia, Rosburia,* and *Faecalibacterium,* suggested a donor-derived benefit [41]. In a secondary analysis of a phase II study of a human microbiota–derived product for treatment of RCDI, Dubberke et al [12] noted that 8 of 11 patients (73%) who were VRE positive at baseline were negative for VRE at the last follow-up stool culture. Using culture-independent techniques, Millan et al [45] demonstrated a significant reduction in the count of antibiotic resistance genes in the stool samples of patients with RCDI with each successive FMT treatment. Notably, not all published cases of RCDI and ARO colonization treated with FMT have demonstrated successful ARO decolonization. Jang et al [39] described a patient with RCDI and VRE stool colonization who was persistently colonized with VRE after 2 FMTs.

## EFFICACY OF FMT FOR ARO DECOLONIZATION AS PRIMARY OUTCOME

Case reports, case series, and prospective studies have also demonstrated the efficacy of FMT for ARO decolonization as a primary outcome (Table 2). These studies were informed by hypotheses of shared risk factors with RCDI and ARO colonization, mouse models, and secondary analyses of patients with RCDI treated with FMT and found to have ARO decolonization.

**Table 2.** Published Case Reports and Series Describing Outcomes of Fecal Microbiota Transplantation for Antibiotic-Resistant Organism Decolonization as Primary End Point

Authors	Patient (s)	Indication	Pretreatment	FMT Donor, No. of FMTs, route	Outcome	Adverse events	Follow-up
Freedman and Eppes [46]	Recurrent otitis media, HLH, osteomyelitis (n = 1)	CP *Klebsiella pneumoniae*; BSI for 5 wk	Polyethylene glycol, omeprazole	Brother, 1, nasoduodenal tube, probiotics for 6 mo	No clinical CP *K. pneumoniae* infection at 1.5 y; 3 stool cultures for CP *K. pneumoniae* negative at 8 mo	None noted	18 mo
Singh et al [47]	Renal transplant recipient (n = 1)	Recurrent ESBL transplant pyelonephritis	“Full colon lavage” without antibiotics	Young, healthy white adult, nasoduodenal tube	ESBL cultures of perineum and throat negative at 1, 2, 4, and 12 wk; rectal cultures negative at 2, 4, and 12 wk though positive at 1 wk; patient able to be relisted for renal transplantation	None noted	3 mo
Lagier et al [48]	n = 1	Asymptomatic stool carriage of OXA-48 *K. pneumoniae* precluded placement in long-term care	Bowel lavage, 4 administrations of colistimethate sodium, gentamicin	Donor not described, 1 50 g of stool), infused by NG tube	Culture negative for carbapenemase-producing *K. pneumoniae* at 7 and 14 d; PCR negative at 7 d	None noted	2 wk
Bilinski et al [49]	Blood disorders (n = 20)	ARO colonization (ESBL, OXA-48, CRE, VRE)	Bowel lavage, PPI, with or without antibiotics	Healthy donor, 1–3 (25 FMTs in 20 subjects), nasoduodenal tube	Complete decolonization in 15/25 patients at 1 mo and in 13/14 at 6 mo	Vomiting, diarrhea	1 mo
Davido et al [13]	Inpatients (n = 8)	CRE or VRE colonization	Bowel lavage, PPI, no antibiotics	Universal donor, 1, nasoduodenal tube	CRE culture negative at 1 and 3 mo in 2/6 patients; VRE culture negative in 1/2 at 3 mo (but not 1 mo)	None noted	3 mo
Dinh et al [15]	Inpatients (n = 17)	CRE or VRE colonization	PPI, bowel lavage, no antibiotics	Healthy donor, 1, nasoduodenal tube	• 3/8 CRE, 3/9 VRE culture negative at 1 mo • 4/8 CRE, 7/8 VRE culture negative at 3 mo	None noted	3 mo
Singh et al [50]	n = 15	ESBL colonization	Bowel lavage, o antibiotics	Healthy donor, 1–2, nasoduodenal tube	Culture was negative at 1 mo in 3/15 patients with 1 FMT and 3/7 with 2 FMTs	Mild discomfort, temporary loose stools	1 mo
Battipaglia et al [51]	n = 10	CRE, VRE, or MDR *Pseudomonas*	Bowel lavage, PPI administered with NG FMTs, enema FMTs requested 2–3-h retention, antibiotics discontinued 48–72 h earlier	Patient-known donors (n = 9) or unrelated donor (n = 1), 1 (n = 7) or 2 (n = 3), enema (n = 8) or NG tube (n = 2)	“Major decolonization” or 3 consecutive negative weekly cultures in 7/10 patients; persistent decolonization (negative at last follow-up) in 6/10; ESBL decolonization noted as secondary outcome in 3/6	Mild, diarrhea in 2 patients, constipation in 1	13-mo median follow-up
Huttner et al [52]	n = 39	ESBL, CRE	Colistin/neomycin for 5 d	Unrelated healthy donors, capsules for 2 d at some centers, NG tube for 1 dose at other centers	ITT: 9/22 patients (41%) in intervention group and 5/17 controls (29%) decolonized; per protocol: 8/16 (50%) in intervention group and 3/13 controls (23%) decolonized	Mild, 4 severe adverse events (1 classified as possibly related to FMT^a^)	5–7 mo
Saïdani et al [53]	n = 10	CPE or CPA	Antibiotics, 2 bowel lavage (×2), PPI, attempted nares decontamination with chlorhexidine		Negative for CPE/CPA at in 8/10 patients at 14 d; 8/15 “FMT success rate” (5 patients had 2 FMTs)		6 mo

Abbreviations: ARO, antibiotic-resistant organism; BSI, blood stream infection, CP, carbapenemase-producing; CPA, CP *Acinetobacter*; CPE, CP Enterobacteriaceae; CRE, carbapenem-resistant Enterobacteriaceae; ESBL, extended-spectrum β-lactamase–producing Enterobacteriaceae; FMT, fecal microbiota transplantation; HLH, hemophagocytic lymphohistiocytosis; ITT, intention to treat; MDR, multidrug-resistant; NG, nasogastric, PPI, proton pump inhibitor, VRE, vancomycin-resistant *Enterococcus*.

^a^The severe adverse event classified as possibly related to FMT was hepatic encephalopathy in a cirrhotic patient.

Multiple case reports have described loss of ARO colonization after treatment with FMT. Freedman and Eppes [46] described their clinical group’s eradication of carbapenem-resistant *Klebsiella pneumoniae* colonization in a 14-year-old girl with hemophagocytic lymphohistocytosis and 5 weeks of persistently positive blood cultures with *K. pneumoniae*. Three follow-up stool cultures over an 8-month period were negative for *K. pneumoniae* and she had no recurrent infections over an 18-month follow-up period [46]. Lagier et al [48] described the successful decolonization of a patient with intestinal colonization with OXA-48 carbapenemase producing *K. pneumoniae*. In both patients, treatment with FMT was motivated by major challenges presented by the ARO colonization. Although these were single case patients without controls, these findings support further testing of hypotheses that ARO decolonization with FMT could reduce ARO infection and improve care for patients with limited options [46, 48].

Bilinski et al [49] reported the results of a prospective study of FMT for ARO colonization in 20 patients with leukemia, multiple myeloma, and thrombotic thrombocytopenic purpura. Efficacy assessments were based on follow-up at 1 week, 1 month, and 6 months after FMT. That study included no control group, and providers were permitted to prescribe antibiotics, as indicated by clinical circumstances. The decolonization end point was met in 15 of 25 FMTs (60%) at 1 month and in 13 of 14 (93%) at 6 months, and *Escherichia coli* was decolonized with more efficacy than *K. pneumoniae* [49]. A subset analysis showed that patients treated with FMT and not prescribed antibiotics were more likely to reach the primary end point of no ARO colonization at 1 month than patients who were prescribed antibiotics [49].

Davido et al [13] reported outcomes of a French multicenter pilot clinical study of FMT for decolonization with CRE and/or VRE. At 1 and 3 months, 2 of 8 patients (25%) and 3 of 8 (38%), respectively, were decolonized [13]. The authors did not identify characteristics that distinguished patients who were decolonized at 1 month from those who were persistently colonized, and no patients who were VRE colonized at baseline were decolonized at 1 month [13]. In a subsequent report from the same multicenter group in France, Dinh et al [15] described similar decolonization proportions of 3 of 8 (38%) and 3 of 9 (33%) among CRE- and VRE-colonized patients, respectively, at 1 week after FMT. At 3 months, 4 or 8 (50%) and 7 of 8 (88%) CRE- and VRE-colonized patients, respectively, were decolonized. In these French studies, no adverse events were reported, and there was no control group to compare the duration of ARO colonization.

Singh et al [50] completed a study of FMT for decolonization of ESBL in 15 patients; when including patients who underwent a second FMT for persistently ESBL-positive cultures, they found an overall decolonization rate of 40%. In their assessment, those authors suggested that differential efficacy between 2 stool donors may have accounted for the differences in outcomes [50].

### Effect of FMT for ARO Decolonization on Need for Contact Isolation

One study comparing 10 patients undergoing FMT to treat ARO colonization and 20 matched retrospective controls demonstrated a decrease of 21.5 days in the median delay to discharge [53]. These authors also reported a median decrease in time to decolonization, from 50.5 to 3 days [53]. Although discharge delays in this study were related to limitations in facilities that were able to receive ARO-colonized patients, these findings suggest that ARO decolonization with FMT could present major cost savings to healthcare systems. They also underscore the potential public health impact of reducing transmission of AROs between patients.

### Effect of FMT on Frequency of Recurrent Infections Other Than RCDI

Studies have also described a potential benefit of breaking the cycle of recurrent urinary tract infections (RUTIs) after FMT for RCDI. Wang et al [44] described an 83-year-old woman with a 25-year history of RUTIs who was treated with FMT for RCDI and had a complete cessation of RUTIs during 25 months of follow-up. A similar observation was reported in a case-control study of 8 patients with ≥3 RUTIs before FMT who were matched to controls with 3 episodes of *C. difficile* infection and ≥3 RUTIs not treated with FMT [43]. Patients with RUTIs treated with FMT were shown to have a decrease in urinary tract infections, from a median of 4 in the year before FMT to 1 in the year after FMT. *E. coli* antimicrobial susceptibilities were noted to improve in the post-FMT setting for cephalosporins, fluoroquinolones, and trimethoprim-sulfamethoxazole [43].

Taken together, these findings show potential efficacy of FMT for decolonization of intestinal colonization with AROs. They have also suggested potential differences in decolonization frequency by ARO type that could be related to pathogen-specific colonization factors. These studies do have important limitations that should be addressed in future studies.

## LIMITATIONS OF PUBLISHED STUDIES ON FMT FOR ARO DECOLONIZATION

Approaches for FMT for ARO decolonization are still early in development. Accordingly, there are still important limitations in our understanding of the safety and efficacy of using FMT for this indication. Most published studies lacked control groups and long-term follow-up periods. They used varying definitions of decolonization and nonstandardized treatment protocols. These limitations restrict the generalizability of the findings. Central questions remain about whether findings of decolonization and decreased frequency of recurrent infections after FMT are directly related to FMT treatments, to other selective pressures such as cessation of antibiotics, or to spontaneous decolonization events. The use of FMT outside healthcare settings, as in travelers returning from locales with higher prevalence of AROs, may also warrant further study.

### Limited Data for Long-Term Safety

A number of FMT case reports and series have described new diagnoses that were temporally associated with the administration of FMT. The intestinal microbiome has also been linked to colorectal cancer, atherosclerosis, and thrombosis [54, 55]. These reports have raised a number of concerns about the possible long-term metabolic, inflammatory and neoplastic risks related to FMT. Long-term prospective cohort studies are needed to further evaluate these potential risks.

### Need for Mechanistic Studies and Control Groups in Clinical Trials of FMT for ARO Decolonization

Preclinical studies have identified mechanisms of microbiome-mediated ARO colonization resistance. For example, in the case of *C. difficile*, bile-salt metabolism, gastrointestinal (GI) luminal pH, and competition for resources are known to be contributing factors in the development of infection [27]. Categories of AROs may occupy distinct spatial niches within the GI tract [56]. Although clinical trials evaluating the efficacy of FMT for treatment of RCDI have had control groups, to date only 1 published prospective clinical trial of FMT for ARO decolonization has included a control group [52]. The lack of a control group greatly weakens estimates of causal associations between FMT and ARO decolonization.

### Need for Further Study of Clinical Failures to Improve Mechanistic Understanding

Many case reports and case series to date have described positive outcomes after FMT. However, the implicit risk of publication bias against negative results of FMT for ARO decolonization should be acknowledged. In 1 of the few studies published with mixed outcomes for FMT ARO decolonization, Stalenhoef et al reported the detailed clinical history, microbiologic, and microbiome analyses for a patient treated with FMT for Verona integron–encoded metallo-β-lactamase–producing *Pseudomonas aeruginosa*. This *Pseudomonas* isolate was not detected in the post-FMT setting, but an ESBL-producing *E. coli* was, which they describe as a clinical success combined with microbiologic failure [57]. They note that their patient had “intact” microbiota diversity before FMT and question the potential efficacy of FMT in patients with normal microbiota diversity.

This potential issue was also observed in 2 negative studies of FMT that were conducted to estimate its efficacy in treating metabolic syndrome and chronic inflammation in virally suppressed patients with human immunodeficiency virus infection [58, 59]. Diversity analyses describe the composition of a microbial community at a high level but do not clarify the functional capacity of these communities. It is also likely that FMT may not be effective for nonintestinal or non–genitourinary tract reservoirs of colonization by ARO (eg, biliary or pulmonary). New analytic tools and databases are being developed that allow analyses of the gene-predicted functional capacity of microbial communities with metagenomic whole-genome sequencing [60]. These tools support moving beyond diversity measures alone and improved understanding of how taxa and their functional capacity may reduce ARO colonization. In turn, this could inform selection of minimal or ideal taxa to include in rationally developed microbiome therapeutics.

## PRACTICAL QUESTIONS AND PRIORITIES TO IMPROVE FUTURE FMT CLINICAL STUDIES

In Table 3, we present a list of challenges, opportunities, and research priorities to improve the current understanding of FMT for ARO decolonization. Although most studies published to date included FMT pretreatments with bowel lavage, with or without antibiotics (Tables 1 and 2), it is not clear whether this step improves efficacy. In a pragmatic study of a lyophilized, encapsulated FMT product taken orally, bowel lavage was abandoned after 4 patients, and the dose was decreased during the study period without a clear compromise in efficacy [61]. These and other practices, such as promotility medications and proton-pump inhibitors coadministered with FMT when delivered via an upper GI tract route or antidiarrheals when administered via an enema, have not been validated in controlled studies. Evidence supporting these practices is generally at the level of expert opinion [37]. These questions warrant additional investigation to improve the safety and efficacy of FMT as it becomes more commonly performed [62, 63].

**Table 3.** Proposed Practical Research Agenda for Future Study of Fecal Microbiota Transplantation for Antibiotic-Resistant Organism Decolonization

Existing Challenge	Recommendations
Wide variability in FMT approaches in published literature	Multicenter clinical trial consortia should be funded to reduce variability in research approaches, improve rigor and reproducibility, and streamline protocol development to study the following prospectively: Ideal feces donor characteristics for ARO decolonization FMT dosing frequency and thresholds for repeating treatment Risks/benefits of bowel-preparation, antibiotic pretreatment Differential effects on specific AROs FMT recipient host factors that modulate FMT efficacy Improve recruiting capacity for rare cases (extreme multidrug resistance) ARO detection in feces in control groups in setting of ongoing antibiotic pressure and varied place of residence Benefits to patients of tailored microbiome therapies of microbial consortia or rationally matched donors
Regulatory future of FMT remains unclear	FDA, industry, and academics should work collaboratively to maintain patient-centered regulatory approaches that balance needs for further study with access to therapies with an immediate need
Unrefined end points of clinical studies	Benchmarking studies are needed to compare the performance characteristics of culture-based, culture-independent, and mixed methods that incorporate both approaches; measures of ARO decolonization should be studied to better estimate precision by number of consecutive swab samples, combining swab samples with PCR- or NGS-based techniques
Limited long-term safety outcomes data	Long-term cohorts and registries are needed to study the long-term safety of microbiome therapeutics

Abbreviations: ARO, antibiotic-resistant organism; FDA, Food and Drug Administration; FMT, fecal microbiota transplantation; NGS, next-generation sequencing; PCR, polymerase chain reaction.

Although a dose of ≥50 g of stool has been recommended, the ideal processing method and the size and frequency of the FMT dose have not been well established [37]. For example, in their study of FMT for ARO decolonization in patients with blood disorders, Bilinski et al [49] noted that none of the patients in their series, treated with 1-day FMT, had complete decolonization. It is important to consider whether stool processing steps are needed to preserve viruses, pH, metabolites, or anaerobic non–spore-forming bacteria. Many studies of FMT efficacy for ARO decolonization have analyzed outcomes with FMT denominators rather than patients. Although the optimal dose, route, preparation, and other FMT details are unresolved, analyzing outcomes with patients as a denominator may clarify the impact of patient-specific factors.

### Regulatory, Ethical, and Practical Considerations

Important questions remain for providers and patients about the use of FMT for ARO decolonization. The regulatory status of FMT remains in flux, but currently the US FDA requires an approved investigational new drug application for all uses of FMT other than RCDI. For RCDI, the FDA has chosen to exercise enforcement discretion. This means that an investigational new drug application is not required but patients should provide consent informed by the discussion of potential risks and the investigational nature of FMT. The novelty of FMT has brought a host of new questions, including whether the material used in FMT is of human origin, akin to a tissue, or if it is a drug that must be consistently manufactured with good manufacturing principles. In addition, naturally occurring substances cannot be patented, which has motivated isolation of variants or derivatives of stool or the active components of FMT to balance drug development costs. On the other hand, patient and FMT-provider advocacy groups have asserted that FMT should remain available in its current form, with access to public stool banks and without explicit FDA approval.

We encourage providers to continue to advocate for patients to ensure access to potentially effective therapies. In the meantime, FMT should not become the standard of care before the safety and efficacy of FMT is rigorously tested in prospective blinded, randomized, controlled trials. Partnership with the FDA for investigational new drug applications, institutional review boards, academics, ethicists, and industry will be necessary for further development of these therapies.

## CONCLUSIONS

The intestinal microbiome is a potentially promising target to directly reduce ARO colonization and possibly subsequent ARO infection. Early evidence suggests that FMT may have the potential to reduce ARO colonization by restoring microbial community composition and function, but further study is necessary. We have outlined a practical research agenda that we believe will improve our current understanding of the safety and efficacy of FMT for ARO colonization and may improve patient care.
