# Corrigendum: The Role of Fecal Microbiota Transplantation in Reducing Intestinal Colonization With Antibiotic-Resistant Organisms: The Current Landscape and Future Directions

**DOI:** 10.1093/ofid/ofz391

**Published:** 2019-10-20

**Authors:** Michael H Woodworth, Mary K Hayden, Vincent B Young, Jennie H Kwon

An error appeared in an article published in the July 2019 issue of the journal [Michael H Woodworth, Mary K Hayden, Vincent B Young, Jennie H Kwon. The Role of Fecal Microbiota Transplantation in Reducing Intestinal Colonization With Antibiotic-Resistant Organisms: The Current Landscape and Future Directions. Open Forum Infect Dis 2019; 6(7);ofz288].

In addition to study support from the National Institutes of Health, the publication was also supported by the Centers for Disease Control and Prevention under Award Number U54CK000481 to MKH.

The authors regret this omission.

